# Assessment of the unwanted tooth movement associated with an extended maxillary fixed retainer (3D analysis)

**DOI:** 10.1186/s12903-024-04622-x

**Published:** 2024-08-06

**Authors:** Mohamed H. Abbas, Essam M. Abdalla, Eiman S. Marzouk, Nadia M. El Harouni

**Affiliations:** Department of Orthodontics, Faculty of Dentistry, Alexandria University, Champollion St., Azarita, P. O. Box: 21521, Alexandria, Egypt

**Keywords:** Superimposition, 3D, Fixed retainers

## Abstract

**Background:**

Posttreatment changes after orthodontic treatment are challenging. One of the main reasons for such a phenomenon is the lack of patient compliance with removable retainers especially in the maxillary arch, due to palatal coverage, deterioration of speech, decreased masticatory efficiency, and loss of retainers. Fixed retainers have been introduced to overcome patient compliance and provide longer stable results. However, teeth still show movements when a six-unit fixed retainer is in place. Thus, in this study, an eight-unit fixed retainer was evaluated in an attempt to eliminate unwanted movements.

**The aim of this research:**

was to assess short-term positional changes associated with an eight-unit extended maxillary fixed retainer.

**Materials and methods:**

A single-arm clinical trial was conducted to address the aim of the study. This research was approved by the institutional review board of the Faculty of Dentistry, Alexandria University (IORG:0008839, No-0479–8/2022). The registration date of this study was 5/06/2023. Twenty-eight patients (19.8 ± 4.5 years) who had finished the active orthodontic phase and started retention had an eight-unit extended maxillary fixed retainer that was bonded to the palatal surface of the maxillary incisors, canines, and the first premolars or the second premolars. Pre-retention and one-year post-retention intra-oral scans were made to produce STL files that were superimposed to determine the amount of tooth change. Additionally, analysis of digital casts and lateral cephalometric radiographs was performed.

**Results:**

Statistically significant changes in all planes and the rotation of teeth after one year of retention were found. The upper right lateral incisor exhibited the most evident change in the vertical plane, while the upper right central incisor exhibited the greatest change overall. Minimal changes in the cast measurements were observed. Lateral cephalometric measurements showed minimal changes after one year of retention, and these changes were not statistically significant except in the interincisal angle and the angle between the upper incisor and the line connecting the A-point to the pogonion.

**Conclusion:**

Increasing the extension of maxillary fixed retainers did not eliminate unwanted tooth movement in the first year of retention.

## Background

Retaining the final position of teeth after orthodontic treatment is crucial for preventing relapse and maintaining long-term results. Failure to implement an effective retention plan can lead to teeth shifting back to their initial positions, which is known as relapse [1]. Additionally, factors such as aging and natural tooth movement can contribute to changes in tooth position even after treatment [2, 3]. To address these concerns, orthodontists commonly employ two types of retainers: removable retainers and fixed retainers (FRs) [4].

The use of removable retainers has proven to be quite efficient in maintaining the position of teeth after orthodontic treatment [5]. However, these appliances require a certain level of compliance from patients [5].

Fixed retainers, which are bonded to the lingual surfaces of teeth, have gained immense popularity in recent years; owing to their aesthetic appeal and ability to ensure retention without relying on patient compliance [6, 7]. In a study published in 2011 [8], 11% of orthodontists favored them in the maxilla.

Fixed retainers can be bonded either to the lingual surface of the canines only or to all six anterior teeth [9]. The choice of which teeth to bond to the retainer has been a topic of debate among orthodontists. However, when comparing the tooth positions after 2 years of retention, it has been observed that canine-to-canine FR results in more relapse in incisors that are not directly bonded to the retainer, compared to a retainer that is attached to all the anterior teeth [10–12]. Thus increasing the number of units attached to the fixed retainer may resist unwanted tooth movement.

Multiple studies [4, 12–18] have shown that there are changes in the position of teeth, that are unrelated to the initial misalignment when using a canine-to-canine (FR) consisting of 6 units. These changes cannot be classified as relapse, but rather as undesired changes. The exact cause of these undesired changes is still being investigated [19]. These movements, occurring within the presence of FR vary from minor rotations of individual teeth to rotation of an entire segment connected to the FR, with the fulcrum located in the incisor region [3, 20, 21].

Unwanted tooth movement with FRs was observed in the vertical dimension, transverse dimension, and sagittal plane. These changes were more evident in maxillary teeth than in mandibular teeth [17, 18]. Furthermore, Isabel Knaup [18] reported that extending the FR from canine to canine without an additional removable retainer resulted in pronounced unwanted tooth movement. They recommended the use of a removable retainer at night to prevent unwanted movements. A growing trend towards dual retention instead of solitary removable or solitary fixed retention is currently being observed to avoid the side effects of canine-to-canine fixed retainers alone, yet this trend is still dependent on patient compliance [22–24].

In cases involving tooth extractions, if the retainer fails to prevent the re-opening of extraction spaces, complications such as periodontal and occlusal problems can occur. Aydin et al. [25] found that increasing the extension of the fixed retainer to the second premolar (8 units) in the mandibular arch without using an additional removable retainer was effective in preserving space closure, with no reported changes in tooth position and no changes in canine position [25–27].

An extended fixed retainer was hypothesized to be a simpler alternative to dual retention protocols while attempting to overcome canine-to-canine FR drawbacks since the number of units to be bonded is increased [25]. The extended fixed retainer was tested only in the mandibular arch in extraction cases, although unwanted movement was found to be more strongly associated with maxillary fixed retainers [25, 26]. There is a lack of studies on extended maxillary fixed retainers in the literature, regarding the extension of the FR and the nature of the associated changes in tooth position.

Therefore, in this study, aiming to study the possibility of eliminating the need for additional removable retainers (dual retention), an eight-unit extended maxillary fixed retainer was bonded directly after finishing the active orthodontic phase, and, the aim of this study was to assess changes in tooth position associated with an eight-unit extended maxillary fixed retainer through 3D superimposition, cast analysis, and lateral cephalometric changes.

## Methods

A single-center prospective open-label single-arm observational study was performed, and the study was registered at Clinicaltrials.gov (NCT05889884). This research was approved by the institutional review board of the Faculty of Dentistry, Alexandria University (IORG:0008839, No-0479–8/2022). The registration date of this study was 5/06/2023. (retrospectively registered) The entire study was conducted at the Orthodontic Department at the Faculty of Dentistry, Alexandria University.

### Inclusion criteria

Patients who had just finished the orthodontic fixed appliance phase (at least one year of treatment) with extraction (four first premolars in Class I severe crowding) or non-extraction treatment and scheduled to start retention.

### Exclusion criteria

Patients who had skeletal palatal expansion or progressive periodontal conditions [28], with general disease and bone disorder or craniofacial syndromes. Patients with any anatomic malformed tooth surface or restoration.

#### Sample size calculation

The sample size was estimated assuming a 5% alpha error and 80% study power. A previous study assessed the 3D post-treatment changes associated with an extended lingual retainer [3]. The total sample size was 26 patients, including an excess of two patients to overcome possible dropouts. The sample size was based on Rosner’s method [29] calculated by G-power 3.0.10 [30].

#### Patient preparation

Before the beginning of the study, the participants and their parents were provided with comprehensive explanations of the study procedures. Informed consent was subsequently obtained from each subject who was enrolled in the study. At the T0 baseline, patients underwent phase one nonsurgical periodontal therapy, which included full mouth supra-gingival and subgingival scaling, root planing, and polishing with eugenol-free paste. Proper oral hygiene instructions were then provided, including the use of a toothbrush, dental floss, and interdental brush, before the FR was bonded.

#### Intervention

An impression was made at T0 to create a removable retainer lest any notable significant changes during the follow-up period could occur, which might require immediate study termination. This retainer could be used to restore the T0 state if necessary (futility point).

An initial scan using CEREC Omnicam (Sirona Dental Systems, Bensheim, Germany) was conducted to generate STL data at T0, representing the dentition before retention.

Pre-retention and post-retention dental casts and lateral cephalometric radiographs were obtained along with full orthodontic records.

## Bonding steps of the extended FR

Several measures have been taken to ensure high bond strength while overcoming the high rate of failure of maxillary FR.

First, pumice polishing was performed for all surfaces to be bonded [31], followed by a sodium hypochlorite swab for 1 min [32] (sodium hypochlorite 5% mint flavor, JK Dental Vision, Egypt) and then acid etching with 36%phosphoric acid for 15 s, followed by rinsing the etchant surface for the same amount of time and gentle drying [33].

**Fig. 1:**
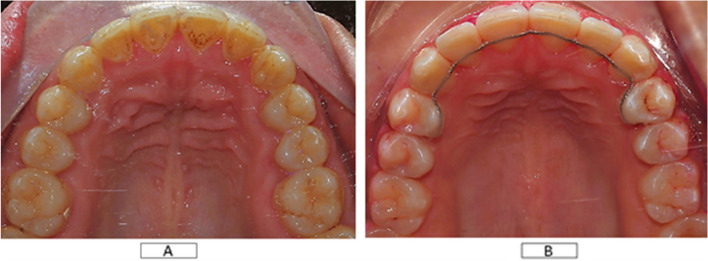
Cropped intra-oral photo for an eight-unit extended maxillary FR

Pre-hydrolyzed no-mix silane primer and silane coupling agents (BISCO PORCELAIN PRIMER, BISCO, USA) were added to all surfaces for bonding [34]. The next step was the application of a bonding agent (ASSURE® PLUS, Reliance Orthodontic Products, USA). Holding of the FR was done with the help of dental floss followed by direct adaptation and festooning of Dead soft wire, 0.027 × 0.011-inch ribbon arch-wire, 8-strand braided wire (FR) (Bond-A-Braid® Lingual Retainer Wire, Reliance Orthodontic Products, USA) from the palatal surface of the right premolar to the left premolar including the palatal surface of all maxillary anterior teeth in passive state away from the line of occlusion (Figure 1). The flowable light-curing composite was applied (Polofil® NHT flowable composite light-curing, Voco, Germany). Curing was carried out for 3 s using high-intensity LED was carried on [35].

After finishing the whole curing process for all the units, excess composite or any interference between FR and lower teeth was selectively ground using articulating paper followed by polishing all the composite surfaces to eliminate any rough area (Fig. 1 A, B).

Details of the oral hygiene instructions were provided, including a thorough explanation of the flossing technique used with the retainer in place, in addition to guidance on utilizing interdental brushes and water flossers.

Patients were asked to urgently contact the operator if any abnormal fracture or detachment in the retainer occurred. Moreover, patients were regularly checked once per month for the whole year to even disclose any detached parts and monitor oral hygiene.

At the 12-month follow-up [13] (T1), all previous records were repeated. This included obtaining a final intra-oral scan to produce a second STL file, which was then superimposed onto the original (baseline T0 STL). This was done to assess the extent of changes that occurred. Additionally, conventional alginate impressions were taken to produce upper and lower casts, and a new lateral cephalometric radiograph was obtained.

### Statistical analysis

Normality was checked for all variables using descriptive statistics (mean, median, and standard deviation), plots (Q-Q plots and histogram), and normality tests. Data from individual teeth were pooled to provide an overall estimate of the amount of tooth movement in each degree of freedom and summarized as mean and standard deviation.

Means and standard deviation were calculated for quantitative variables, while frequencies and percentages were calculated for qualitative variables. Comparisons of quantitative variables at T0 and T1 were done using paired t-test and Wilcoxon signed ranks test according to the variable normality. Mean difference and 95% confidence intervals (CIs) were calculated. Comparisons of qualitative variables at T0 and T1 were performed using the McNemar test. Significance was set at a *p*-value < 0.05. The data were analyzed using IBM SPSS for Windows (version 26.0).

## Reliability tests

All cast measurements, STL files superimposition, and lateral cephalometric radiograph tracing were performed by two different examiners. Inter-examiner reliability was calculated and the intraclass correlation coefficient (ICC) ranged from 0.90 to 0.94 indicating good to excellent agreement between the examiners [36].

All measurements of casts, cephalometric analysis, and STL superimposition were documented twice independently on two separate occasions with an interval of 2 weeks by the same examiner. The intraclass correlation coefficient (ICC) ranged from 0.89 to 0.948.

### Methods of assessment

#### 3D superimposition

A comparison of 3D surface models before and after retention was performed by superimposing the two STLs on the medial part of the third rugae and a small region dorsal to it [37–39]. Superimposition was performed by MEDIT Link 3.1.0 and MEDIT design 2.1.3 software along with OnDemand3D version1.0

To evaluate tooth movement, five points are identified on each tooth: the mesial and distal points of the occlusal surface, the gingival and occlusal boundaries of the buccal surface, The functional Axis of the Clinical Crown (FACC) (long axis), and the gingival boundary of the lingual FACC. The occlusal points for incisors are represented by the incisal edge, while for canines, it is the canine ridges’ incisal tip. The buccal and lingual FACCs of incisors, canines, and premolars are determined by identifying lines passing through the highest contour of the buccal surfaces and projecting them onto the lingual surfaces. The gingival and occlusal limits of the buccal and lingual FACCs are used to define the lingual FACC, and these points are digitized. Cross-sections of each tooth are made from the centroid point (middle of the incisal edge) to analyze buccolingual changes when viewing the tooth from a proximal perspective [38].

#### Linear measurement assessment [38]

##### Bucco-lingual assessment

**Fig. 2:**
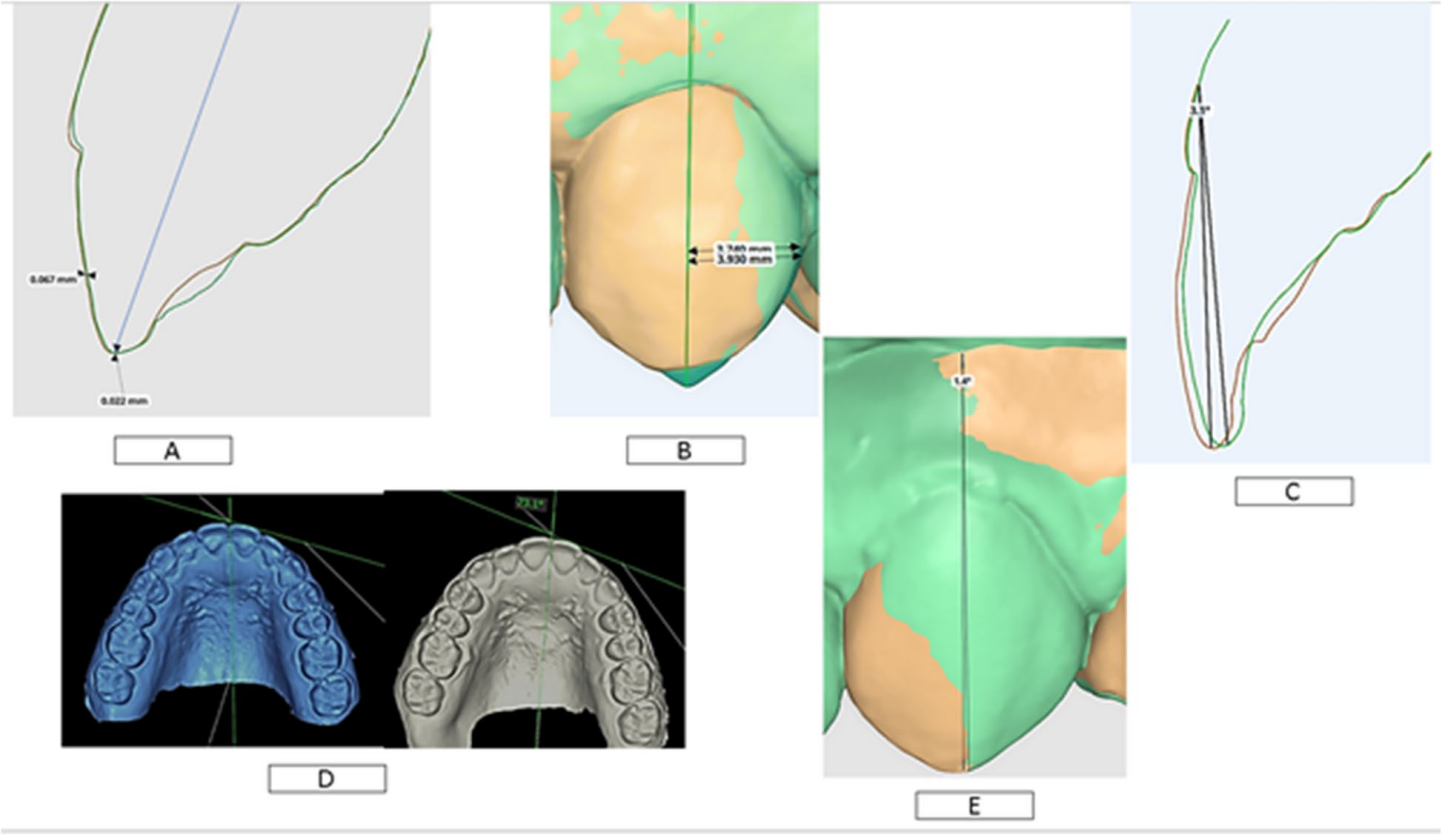
**A** Superimposition for linear changes in labiolingual and occluso-gingival direction. **B** 3D-superimposition for linear changes in mesiodistal direction. **C** 3D-superimposition for angular changes in torque. **D** Angle of rotation. **E** 3D-superimposition for angular changes in tip

This was performed by having a cross-sectional cut for each tooth on superimposed STL files and measuring the linear distance from the highest contour of the tooth to the line representing the long axis of the tooth(T0-T1) when it was seen proximally. (Fig. 2A).

##### Occluso-gingival movement assessment

This was performed by having a cross-sectional cut for each tooth on superimposed STL files and measuring the subtraction linear distance from the tip of the two incisal edges (lowest point of incisal edge) of both (T0-T1). (Fig. 2A).

##### Mesio-distal assessment

This was performed by subtracting the linear distance from the disto-fascial-incisal point angle to the line representing the long axis of each tooth (T0-T1) in the (pre-post retention superimposed STL file) (Fig. 2B).

#### Angular measurements [38]

##### Torque assessment

This was performed by having a cross-sectional cut for each tooth on superimposed STL files and measuring the angle between the long axis of each tooth (pre-post retention) when each tooth was seen proximally (Fig. 2C).

##### Rotational angle

This was calculated by measuring the angle between the tangent line to the highest facial contour of each tooth and the mid-palatal line of the maxillary arch for each STL file (pre and post-retention). Then, the two angles are subtracted to determine the amount of rotation for each tooth [40] (Fig. 2D).

##### Tip assessment

This was performed by measuring the angle between the long axis of each tooth (pre-post retention superimposed STL file) when each tooth was seen facially (Fig. 2E).

### Cast analysis

Casts were analyzed to compare changes from the baseline T0 (STL) file and the final (STL)file.

#### Inter-canine width

Linear horizontal distance between the cusp tip points of the right and left canines in the maxillary arch [41].

#### Inter-premolar width

Linear horizontal distance from the first premolar of the left side to the right side at the distal end of the occlusal groove [42].

#### Inter-molar width

The linear horizontal distance between the maxillary left permanent molar and the right at its mesial pit on the occlusal surface [42].

#### Overbite

Vertical overlap of the maxillary incisors over the mandibular incisors [41].

#### Overjet

Horizontal distance between the labial surfaces of the maxillary incisors and mandibular incisors [41].

#### The irregularity index

Is a quantitative method for assessing anterior irregularity. The linear displacement of the adjacent anatomic contact points of the incisors was determined, and the sum of the five measurements for the 6 anterior teeth represented the irregularity index value of the patient [43]. The index scores are as follows:

**Table:** 

0 Perfect alignment	l-3 Minimal irregularity
4-6 Moderate irregularity	7–9 Severe irregularity
10 Very severe irregularity	

### Lateral cephalometric analysis

The changes in the axial inclination of upper incisors before bonding the upper maxillary extended fixed retainer and after one year of bonding through the following measurements.

**Table:** 

A-Upper incisor (UI) to NA (linear and angular)	B- (UI) to FH
C-(UI) to palatal plane	D- (UI) to occlusal plane
E- (UI) to SN plane	F- (UI) to (LI) (interincisal angle)
G- (UI) to A/Pog (linear measurements)	

## Results

The demographic data for all patients (*n* = 28) are shown in Table 1. Measurements for various cast measurements showed insignificant changes. (Table 2) Overbite, overjet, inter-canine width, inter-premolar width, and inter-molar width showed no significant differences throughout the follow-up time. The irregularity index showed a more noticeable change, increasing from 0.00 at T0 to 0.18 at T1, with a difference of 0.18 (SD 0.48). This change approached statistical significance (*p* = 0.06). Only the irregularity index showed a potential increase, but it was not statistically significant (*p* = 0.06).

**Table 1:** Sample description (*n* = 28)

**Age**	**Mean (SD)**	19.82 (3.33)
**Gender: n (%)**	**Male**	6 (21.4%)
	**Female**	22 (78.6%)
**Treatment technique: n (%)**	**Extraction**	11 (39.3%)
	**Non-extraction**	17 (60.7%)

Demographic data of the sample

*SD* Standard deviation, *n* Frequency

**Table 2:** Cast measurements pre- and post-retention

	**Pre-retention (T0)**	**Post-retention (T1)**	**Difference (T1-T0)**	**95% CI**	***P* value**
	**Mean (SD)**				
**Intercanine width**^a^	35.62 (1.92)	35.82 (1.92)	0.20 (0.72)	-0.08, 0.47	0.16
**Interpremolar width**^a^	37.80 (1.70)	37.66 (1.64)	-0.14 (0.56)	-0.36, 0.08	0.21
**Intermolar width**^a^	44.40 (2.63)	44.53 (2.74)	0.12 (0.74)	-0.16, 0.41	0.39
**Irregularity index**^b^	0.00 (0.00)	0.18 (0.48)	0.18 (0.48)	-0.006, 0.36	0.06
**Overbite**^a^	2.43 (0.50)	2.39 (0.50)	-0.04 (0.19)	-0.11, 0.04	0.33
**Overjet**^a^	2.04 (0.19)	2.07 (0.26)	0.03 (0.19)	-0.04, 0.11	0.33

*SD* Standard deviation, *CI* Confidence interval

^a^Paired samples t-test

^b^Wilcoxon signed ranks test

^*^Statistically significant at *p* value < 0.05

All pre- and post-retention lateral cephalometric measurements were performed (Table 3) and showed statistically insignificant changes except for the inter-incisal angle which showed a significant decrease of -1.50 (SD: 3.61) (*p*-value: 0.04).

**Table 3:** Lateral cephalometric measurements pre- and post-treatment

	**Pre-retention (T0)**	**Post-retention (T1)**	**Difference (T1-T0)**	**95% CI**	***P* value**
	**Mean (SD)**				
**U1/NA (mm)**	5.57 (2.66)	5.71 (2.80)	0.14 (1.24)	-0.34, 0.62	0.55
**U1/NA (angle)**	21.68 (5.66)	22.30 (5.62)	0.63 (2.10)	-0.19, 1.44	0.13
**U1/FH**	113.86 (6.11)	113.93 (6.23)	0.07 (3.16)	-1.15, 1.30	0.91
**U1/Pal**	67.39 (6.64)	67.64 (7.07)	0.25 (2.59)	-0.76, 1.26	0.61
**U1/Occ**	59.96 (5.69)	60.36 (5.60)	0.39 (2.96)	-0.76, 1.54	0.49
**U1/SN**	102.71 (6.97)	103.43 (6.70)	0.71 (1.98)	-0.05, 1.48	0.07
**U1/L1**	126.54 (9.10)	125.04 (7.91)	-1.50 (3.61)	-2.90, -0.10	0.04^*^
**U1/A-POG**	8.50 (2.50)	8.88 (2.54)	0.36 (1.06)	-0.04, 0.79	0.07

*SD* Standard deviation, *CI* Confidence interval

^*^Statistically significant at *p* value < 0.05

Linear changes in (Table 4), For the mesiodistal linear measurements, all teeth showed statistically significant changes. The mean increase in mesiodistal position changes ranged from 0.13 to 0.22 across the different teeth. The highest change in mesiodistal direction was seen in tooth #22, with a mean increase of 0.22 (SD: 0.16) (*p*-value: < 0.001). Conversely, the smallest change in mesiodistal direction was found in tooth #13, which had a mean increase of 0.13 (SD: 0.24) (*p*-value: 0.009).

**Table 4:** Mesiodistal, faciolingual, occlusogingival ( linear measurements changes)

	Tooth	Pre-retention	Post-retention	Difference (changes)	95% CI	*P* value
		Mean (SD)				
Mesiodistal	#14	3.97 (0.49)	4.13 (0.54)	0.16 (0.19)	0.09, 0.24	< 0.001^*^
	#13	4.69 (0.54)	4.82 (0.55)	0.13 (0.24)	0.04, 0.22	0.009^*^
	#12	3.71 (0.44)	3.87 (0.45)	0.16 (0.19)	0.08, 0.23	< 0.001^*^
	#11	4.57 (0.48)	4.75 (0.47)	0.18 (0.18)	0.11, 0.25	< 0.001^*^
	#21	4.76 (0.51)	4.92 (0.51)	0.17 (0.19)	0.09, 0.24	< 0.001^*^
	#22	4.03 (0.50)	4.24 (0.51)	0.22 (0.16)	0.16, 0.28	< 0.001^*^
	#23	4.48 (0.45)	4.67 (0.47)	0.19 (0.26)	0.08, 0.28	0.001^*^
	#24	3.79 (0.43)	3.93 (0.46)	0.15 (0.18)	0.08, 0.21	< 0.001^*^
Faciolingual	#14	4.30 (0.62)	4.13 (0.54)	0.17 (0.15)	0.11, 0.23	< 0.001^*^
	#13	5.01 (0.59)	4.82 (0.55)	0.19 (0.15)	0.13, 0.25	< 0.001^*^
	#12	4.13 (0.52)	3.87 (0.45)	0.26 (0.17)	0.19, 0.33	< 0.001^*^
	#11	5.04 (0.57)	4.75 (0.47)	0.29 (0.20)	0.22, 0.37	< 0.001^*^
	#21	5.18 (0.52)	4.92 (0.51)	0.26 (0.13)	0.21, 0.31	< 0.001^*^
	#22	4.43 (0.53)	4.24 (0.51)	0.19 (0.13)	0.14, 0.24	< 0.001^*^
	#23	4.83 (0.51)	4.67 (0.47)	0.16 (0.18)	0.10, 0.23	< 0.001^*^
	#24	4.14 (0.51)	3.93 (0.46)	0.21 (0.16)	0.15, 0.27	< 0.001^*^
Occlusogingival		Intrusion		Extrusion		
		N (%)	Mean (SD) mm	N (%)	Mean (SD) mm	
	#14	9 (32%)	-0.41 (0.35)	19 (68%)	0.20 (0.23)	
	#13	12 (42.9%)	-0.41 (0.35)	16 (57.1%)	0.24 (0.25)	
	#12	10 (35.7%)	-0.35 (0.21)	18 (64.3%)	0.34 (0.45)	
	#11	14 (50%)	-0.48 (0.24)	14 (50%)	0.32 (0.52)	
	#21	13 (46.4%)	-0.41 (0.26)	15 (53.6%)	0.26 (0.29)	
	#22	11 (39.3%)	-0.41 (0.22)	17 (60.7%)	0.21 (0.15)	
	#23	11 (39.3%)	-0.30 (0.15)	17 (60.7%)	0.20 (0.20)	
	#24	12 (42.9%)	-0.42 (0.71)	16 (57.1%)	0.22 (0.19)	

Paired t-test was used

*SD* Standard deviation, *CI* Confidence interval

^*^Statistically significant at *p* value < 0.05

Regarding the faciolingual changes measurements, similar findings were observed. All teeth exhibited statistically significant changes, The mean increase in faciolingual direction ranged from 0.16 to 0.29 across the different teeth, with the highest change occurring in tooth #11. with a mean increase of 0.29 (SD: 0.20) (*p*-value: < 0.001). On the other hand, tooth #23 demonstrated the smallest change in faciolingual plane, with a mean increase of 0.16 (SD: 0.18) (*p*-value: < 0.001). In terms of occlusogingival changes, the data presented the number (N) and percentage (%) of teeth exhibiting intrusion or extrusion. It can be observed that the majority of teeth experienced extrusion post-retention. The teeth with the highest percentage of extrusion were #14 and #12, with 68% and 64.3%. Conversely, the teeth with the lowest percentage of extrusion were #11 and #21, at 50% and 53.6%, respectively.

Teeth experienced minimal changes in rotational angle after retention Table 5, the teeth displayed small differences with no statistically significant findings.

**Table 5:** Angular measurements changes

	**Rotational angle**					**Angular measurements**	
**Tooth**	**Pre-retention**	**Post-retention**	**Difference (change)**	**95% CI**	***P* value**	**Torque**	**Tip**
	**Mean (SD)**					**Mean (SD)**	
**#14**	31.39 (5.59)	31.10 (6.48)	0.29 (3.76)	-1.16, 1.75	0.68	1.73 (0.87)	1.47 (0.62)
**#13**	38.73 (5.81)	39.12 (6.16)	-0.38 (5.99)	-2.71, 1.94	0.74	1.83 (0.95)	1.76 (1.12)
**#12**	56.46 (5.54)	56.99 (6.25)	-0.53 (3.54)	-1.90, 0.84	0.44	2.10 (1.26)	1.55 (0.79)
**#11**	77.44 (3.66)	78.14 (4.62)	-0.70 (4.13)	-2.30, 0.90	0.38	2.32 (1.09)	1.62 (0.87)
**#21**	74.32 (2.47)	73.89 (3.24)	0.43 (2.94)	-0.71, 1.57	0.44	2.49 (1.26)	1.90 (0.81)
**#22**	55.63 (4.93)	55.36 (5.80)	0.27 (3.92)	-1.25, 1.79	0.72	2.38 (1.42)	1.48 (0.55)
**#23**	39.18 (4.15)	38.64 (4.42)	0.54 (4.26)	-1.11, 2.19	0.51	1.99 (1.19)	1.54 (0.61)
**#24**	30.53 (4.42)	31.17 (4.10)	-0.64 (4.17)	-2.25, 0.98	0.43	1.71 (1.11)	1.80 (0.88)

Paired t-test was used

Angular changes in tip and torque showed that there were variations in both torque and tip angular measurements among the different teeth. For torque changes, tooth #22 had the highest mean value of 2.49 (SD 1.26), followed closely by tooth #22 with a mean of 2.38 (SD 1.42). On the other hand, tooth #24 had the lowest mean torque change at 1.71 (SD 1.11). In terms of tip changes, tooth #21 exhibited the largest mean value at 1.90 (SD 0.81), while, tooth #14 showed the lowest mean tip change at 1.47 (SD 0.6).

On comparing the whole changes in 3D among teeth, Table 6 showed that there were variations in the linear measurements among the different teeth. Among the linear measurements, it is evident that the occluso-gingival movement, particularly in teeth #12, #11, #21, and #23, showed statistically significant differences. This suggests that the vertical movement (occluso-gingival) was more pronounced in these teeth compared to the mesiodistal and facio-lingual movements.

**Table 6:** Comparison of linear measurements

	**Superimposition results**			
**Tooth**	**Mesiodistal**	**Faciolingual**	**Occlusogingival**	***P* value**
	**Mean (SD)**			
**#14**	0.16 (0.19)	0.17 (0.15)	0.27 (0.29)	0.87
**#13**	0.13 (0.24)	0.19 (0.15)	0.31 (0.30)	0.054
**#12**	0.16 (0.19)^**a**^	0.26 (0.17)^**b**^	0.35 (0.38)^**b**^	0.009^*^
**#11**	0.18 (0.18)^**b**^	0.29 (0.20)^**b**^	0.40 (0.40)^**b**^	0.03^*^
**#21**	0.17 (0.19)^**a**^	0.26 (0.13)^**a**,**b**^	0.33 (0.29)^**b**^	0.03^*^
**#22**	0.22 (0.16)	0.19 (0.13)	0.29 (0.20)	0.78
**#23**	0.19 (0.26)^**a**^	0.16 (0.18)^**a**,**b**^	0.24 (0.19)^**b**^	0.01^*^
**#24**	0.15 (0.18)	0.21 (0.16)	0.31 (0.48)	0.18

Friedman test was used

^a^^,^^b^Different letters denote statistically significant differences between groups using Bonferroni adjustment

^*^Statistically significant at *p* value < 0.05

On comparing linear measurements and angular measurements Table 7, in terms of mesiodistal changes, no statistically significant differences were observed among the teeth. Similarly, for facio-lingual changes, no significant differences were found either. However, when analyzing the angular measurements, statistically significant differences were found in the mean values of teeth #12, #11, #21, and #22. This suggests that these teeth exhibited notable differences in terms of their angulation compared to the other teeth studied. Additionally, the Wilcoxon signed ranks test indicated that teeth #12, #11, #21, and #22 showed statistically significant differences in angular measurements, with distinct angulation pattern changes compared to the other teeth.

**Table 7:** Comparison of linear measurements and angular

	**Tooth**	**Tip**	**Torque**	***P* value**
		**Mean (SD)**		
**Linear measurements**	**#14**	0.16 (0.19)	0.17 (0.15)	0.74
	**#13**	0.13 (0.24)	0.19 (0.15)	0.82
	**#12**	0.16 (0.19)	0.26 (0.17)	0.01^*^
	**#11**	0.18 (0.18)	0.29 (0.20)	0.01^*^
	**#21**	0.17 (0.19)	0.26 (0.13)	0.13
	**#22**	0.22 (0.16)	0.19 (0.13)	0.55
	**#23**	0.19 (0.26)	0.16 (0.18)	0.10
	**#24**	0.15 (0.18)	0.21 (0.16)	0.09
**Angular measurements**	**#14**	1.73 (0.87)	1.47 (0.62)	0.10
	**#13**	1.83 (0.95)	1.76 (1.12)	0.11
	**#12**	2.10 (1.26)	1.55 (0.79)	0.04^*^
	**#11**	2.32 (1.09)	1.62 (0.87)	0.01^*^
	**#21**	2.49 (1.26)	1.90 (0.81)	0.03^*^
	**#22**	2.38 (1.42)	1.48 (0.55)	0.01^*^
	**#23**	1.99 (1.19)	1.54 (0.61)	0.11
	**#24**	1.71 (1.11)	1.80 (0.88)	0.60

Wilcoxon signed ranks test was used

^*^Statistically significant at *p* value < 0.05

## Discussion

Orthodontic fixed retainers have been widely regarded as an essential component of orthodontic treatment, maintaining dental occlusion, stability and preventing relapse [5]. The most commonly used type of fixed retainer is the canine-to-canine FR retainer, which is bonded to all anterior teeth on both sides [9]. Studies have documented various types of tooth movement that can occur with a canine-to-canine fixed retainer, even while the retainer is in place. These movements can range from minor rotations of individual teeth to the rotation of an entire segment that is connected with the fixed retainer, with a fulcrum in incisors [3, 13, 14].

The authors’ hypothesis was addition of an extra two units to 6-unit FR would increase the root surface area of the bonded segment resisting unwanted tooth movement, and based on 3D superimposition quantitative measurement of the unwanted tooth movement can be determined.

Superimposition was done using the area of the medial part of the third rugae and a small dorsal region to it as it is considered a high anatomical stability to provide a stable, reliable superimposition [39, 44, 45].

Based on this study’s findings despite the increase in extension of FR, positional changes were evident among teeth, The most evident change in the vertical plane was extrusion. The irregularity index showed significant changes that suggest that there was an increase in teeth irregularity after 12 months of retention. Lateral cephalometric changes were significant only with U1/L1 after 12 months of retention. This suggests that there was a decrease in the angle between the upper incisor (U1) and the lower incisor (L1) after retention which may be due to gross changes in the lower teeth rather than the upper teeth since most of the upper teeth reading showed minimal or no significant changes. Among the teeth, tooth #12 had the highest change in rotational angle, with a difference of -0.53 degrees (SD 3.54). However, this change was not statistically significant (*p*= 0.44), suggesting that the rotational angle remained relatively stable after retention. However, some findings were statistically significant yet clinically insignificant since there was no clinical impact on occlusion and no gross reported changes by the patient in the first 12 months of retention. However. Long-term follow-up studies are needed to test the long-term severity of unwanted changes along with its failure rate, Although PDL has recoil memory, which is one the main factors responsible for tooth movement, it takes 3-months up to 8 months in some cases to reorganize in the final position achieved in active orthodontic phase, therefore the maximum impact of relapse occurs in average in the first 3 to 8 months after finishing the treatment [29, 30, 46].

The addition of an extra two units to a 6-unit FR was challenging since a 6-unit FR is known to have a high failure rate [47]. Several precautions were carried on to overcome this problem. To ensure high bond strength several measures have been taken to provide that. Starting with pumice polishing enhances shear bond strength [31]. Followed by Sodium hypochlorite 5% swab for 1 min to all surfaces to be bonded to enhance bond strength and remove organic pellicle of dental plaque [32]. Pre-hydrolyzed silane primer was added to etched surfaces to enhance the bonding strength [34]. Patients were instructed to regularly follow the integrity of the retainer while brushing, avoid any extra hard food that might break the retainer and if any loss of integrity took place, the patient must immediately ask for an appointment to fix it. Despite using several measures to decrease the failure rate, two cases have experienced breakage of the FR, and the patients presented for repair with the same bonding protocol after removal of the composite attached to the broken parts and tooth without compromising retainer integrity.

Our results agree with multiple studies that have provided evidence of teeth positional changes with a canine-to-canine fixed retainer that are unrelated to the original malocclusion [4, 12, 15–20]. These changes are considered undesired movement rather than relapse [48], and the exact cause behind them remains an area of ongoing research [21]. Klaus et al., found that Maxillary retainers (from canine to canine) were affected more often than the mandibular retainers with unwanted tooth movement, which was one of the motives for performing this study in the maxillary arch besides other reasons which are related to compliance to removable retainers in the upper arch [49]. This follows the results of Knaup et al., [15, 16] who reported more unwanted tipping movement with upper teeth than lower ones. Unwanted tooth movement was pronounced with a fixed retainer extending from canine to canine only without an additional removable retainer. To avoid unwanted movement of the canine, nightwear of a removable retainer was recommended.

Many authors [17, 50, 51] reported many other complications such as space re-opening, change in labiolingual inclination of the anterior segment, change in the mesiodistal tip of anterior teeth crowns, gingival recession, the black triangle between incisors in both extraction and non-extraction cases. Most of these changes were related to canines and torque changes, this matched the results of Shaughnessy [18] who found torque discrepancy between adjacent teeth, especially for the distal end of the retainer (canines) which was also found by Maria Francesca Sfondrini [52]. Canines therefore were the most frequent site of unwanted tooth movement, some authors reported severe forms of complications ranging from intrusion of maxillary canine bonded with FR to complete avulsion of the canine [53]. Therefore increasing extension to premolars was carried on in this study in maxillary teeth aiming to overcome unwanted changes. However, Further future RCT studies with a control group are needed to compare changes in teeth position between the removable retainer group, conventional 6-unit FR, and extended 8-unit FR. Indirect retainer fabrication (3D printed or conventionally indirect fabricated FR will need to be tested against a directly bonded extended retainer. Future studies are needed with specific groups (extraction or non-extraction). To enhance the validity and generalizability of results future studies with higher study power will be needed.

## Conclusion

Increasing the extension of maxillary FRs does not prevent unwanted tooth movement.

In short-term follow up unwanted tooth movement with an extended maxillary FR was statistically significant but not clinically significant.

## Data Availability

The datasets used during the current study are available from the corresponding author upon reasonable request.
